# A multi-view multimodal deep learning framework for Alzheimer's disease diagnosis

**DOI:** 10.3389/fnins.2025.1658776

**Published:** 2025-10-01

**Authors:** Jianxin Feng, Xinyu Zhao, Zhiguo Liu, Yuanming Ding, Feng Wang

**Affiliations:** ^1^Communication and Network Key Laboratory, Dalian University, Dalian, China; ^2^School of Information Engineering, Dalian University, Dalian, China; ^3^Dalian University Affiliated Xinhua Hospital, Dalian University, Dalian, China

**Keywords:** Alzheimer's disease, multimodal fusion, Multi-view learning, cross-modal attention, neuroimaging

## Abstract

**Introduction:**

Early diagnosis of Alzheimer's disease (AD) remains challenging due to the high similarity among AD, mild cognitive impairment (MCI), and cognitively normal (CN) individuals, as well as confounding factors such as population heterogeneity, label noise, and variations in imaging acquisition. Although multimodal neuroimaging techniques like MRI and PET can provide complementary information, current approaches are limited in multimodal fusion and multi-scale feature aggregation.

**Methods:**

We propose a novel multimodal diagnostic framework, Alzheimer's Disease Multi-View Multimodal Diagnostic Network (ADMV-Net), to enhance recognition accuracy across all AD stages. Specifically, a dual-pathway Hybrid Convolution ResNet module is designed to fuse global semantic and local boundary information, enabling robust three-dimensional medical image feature extraction. Furthermore, a Multi-view Fusion Learning mechanism, which comprises a Global Perception Module, a Multi-level Local Cross-modal Aggregation Network, and a Bidirectional Cross-Attention Module, is introduced to efficiently capture and integrate multimodal features from multiple perspectives. Additionally, a Regional Interest Perception Module is incorporated to highlight brain regions strongly associated with AD pathology.

**Results:**

Extensive experiments on public datasets demonstrate that ADMV-Net achieves 94.83% accuracy and 95.97% AUC in AD versus CN classification, significantly outperforming mainstream methods. The framework also shows strong discriminative capability and excellent generalization performance in multi-class classification tasks.

**Discussion:**

These findings suggest that ADMV-Net effectively leverages multimodal and multi-view information to improve the diagnostic accuracy of AD. By integrating global, local, and regional features, the framework provides a promising tool for assisting early diagnosis and clinical decision-making in Alzheimer's disease. The implementation code is publicly available at https://github.com/zhaoxinyu-1/ADMV-Net.

## 1 Introduction

Alzheimer's disease (AD) is an irreversible neurodegenerative disorder (Hu et al., 2023) characterized primarily by memory decline, cognitive impairment, and loss of daily living abilities. With the acceleration of global aging, the number of AD patients is rapidly increasing, with projections indicating that the global patient population will reach 139 million by 2050, imposing substantial economic and psychological burdens on both society and individuals (Alzheimer's Disease, 2023). Early diagnosis is crucial for delaying disease progression, improving patients' quality of life, and reducing stress on families and society.

Neuroimaging techniques such as structural magnetic resonance imaging (sMRI) and positron emission tomography (PET) can capture abnormal changes in the brain (Damulina et al., 2020), making them essential tools for AD diagnosis. In recent years, deep learning technologies have achieved breakthroughs in this field, particularly excelling in multimodal data processing. Under end-to-end training (Choudhury et al., 2024), these techniques leverage neural network backpropagation to learn data-driven representations associated with pathology, thereby reducing the need for manual feature engineering. In AD diagnosis research, single-modal approaches suffer from limited information and cannot comprehensively reflect the complexity of the disease, resulting in insufficient sensitivity and specificity for early identification and precise diagnosis (Fjell et al., 2010). Consequently, multimodal approaches have emerged as an effective solution. By integrating information from different modalities such as sMRI and PET, these methods can more comprehensively reflect pathological changes and improve diagnostic accuracy (Chen et al., 2023).

In recent years, multimodal feature fusion has achieved significant progress in neuroimaging analysis. Sparse graph optimization (Liang et al., 2024) and transfer learning (Ramani et al., 2024) Wu et al. (2022)) have improved classification performance, while integration of cognitive tests with genetic factors (Zhang et al., 2024), refined ROI selection (Lei et al., 2024), and volumetric segmentation (You et al., 2023) have enhanced analytical precision. Tang et al. (2024a)) proposed CEFM combined with ECSA, which significantly enhanced AD feature recognition capability, and Jia et al. (2024)) constructed a multimodal global-local fusion framework that effectively integrated clinical tabular data with MRI information. MLCA (Wan et al., 2023), CBAM (Woo et al., 2018), and slice-level (Chen et al., 2022), multi-patch (Ye et al., 2024), and 3D multi-head attention (Huang et al., 2024) mechanisms have improved model sensitivity to important features by focusing on key regions. Cross-modal long-range dependency modeling based on Transformers (Tang et al., 2024b)(Alinsaif, 2025) has enriched multi-scale feature representation, while harmonic wavelet regional pyramids (Liu et al., 2023b), kernel attention fusion (Pei et al., 2022), and the combination of self-attention pooling with graph convolution (Sang and Li, 2024) have enhanced perception of complex patterns. Dynamic balancing strategies [HAMF (Lu et al., 2024), WMCL-HN (Yu et al., 2025)] and multi-scale convolutional ensemble learning (Yan et al., 2025) have optimized model performance, and BiFPN (Tan et al., 2020) has achieved efficient fusion of hierarchical features from different modalities. However, most methods still rely on simple concatenation or weighting and fail to fully exploit complementary information between modalities, leaving room for improvement in fusion depth.

To address the aforementioned issues, this paper proposes a multi-view multimodal Alzheimer's disease diagnostic model—ADMV-Net. This framework first extracts global semantic and local boundary features in parallel from sMRI and PET images through a dual-pathway Hybrid Convolution ResNet (HCNet). Subsequently, we design a Multi-view Fusion Learning (MVFL) mechanism to capture complementary information from global, local, and latent views, significantly enhancing feature representation. Finally, we utilize a Regional Interest Perception Module (RIPM) to construct a brain region weight matrix that identifies key brain regions associated with Alzheimer's disease. The main contributions of this paper include:

1. We propose a novel multi-view multimodal fusion model that effectively integrates three-dimensional imaging data from PET, GM, and WM modalities to improve diagnostic accuracy for AD.
2. We introduce a dual-path feature extraction structure, HCNet, which achieves efficient fusion of global semantic and local boundary information, thereby improving the feature representation of three-dimensional medical images.
3. We design a multi-view fusion learning module, MVFL, which captures diverse features from multiple perspectives through global, local, and latent learning modules, further strengthening feature representation.
4. We used a brain region weight matrix to learn the importance of different brain regions.

## 2 Materials and methods

### 2.1 Dataset and preprocessing

The study utilized paired T1-weighted MRI and PET scan data from the Alzheimer's Disease Neuroimaging Initiative (ADNI) (Petersen et al., 2010) and the Australian Imaging, Biomarkers and Lifestyle Study of Ageing (AIBL) (Selvaraju et al., 2017) databases. The ADNI database included 339 participants with Alzheimer's Disease (AD), 473 participants classified as Cognitively Normal (CN), and 525 participants identified with Mild Cognitive Impairment (MCI), with female ratios of 50.16%, 49.28%, and 52.6%, and average ages of 75.23, 74.98, and 75.11 years, respectively. The AIBL study comprised 82 participants with AD, 105 participants with CN, and 95 participants with MCI, with female ratios of 50.36%, 51.24%, and 49.99%, and average ages of 73.56, 72.26, and 74.41 years, respectively. All subjects underwent both sMRI and PET examinations, with the ADNI dataset specifically including FDG-PET and PIB-PET imaging.

We employed SPM and CAT tools to perform rigorous preprocessing of MRI and PET data to ensure quality consistency. MRI preprocessing included unified voxel resampling, slice timing correction, head motion correction, normalization to standard space, and tissue segmentation (GM, WM, and CSF), followed by extraction of GM and WM. The PET data preprocessing pipeline involved MRI alignment, spatial normalization, skull stripping, and smoothing to optimize signal quality. Based on functional relevance considerations, we used the AAL116 template to divide the whole brain into 116 anatomically and functionally defined ROIs and selected the first 90 ROIs (excluding cerebellar regions) as the final analysis targets.

### 2.2 Experimental setup and evaluation metrics

Our experiments were conducted in a Linux environment equipped with dual NVIDIA RTX 4090 GPUs and 120GB memory, implemented using Python 3.10 and Torch 2.2.0. Model training employed a stochastic gradient descent optimizer with an initial learning rate of 0.001 and dynamic adjustment using a cosine annealing strategy. Network training parameters were set with a batch size of 16 and a total of 40 training epochs.To assess the statistical significance of performance differences between methods, we conducted paired t-tests for all comparative experiments.

Models were trained separately for three classification tasks (AD vs CN, AD vs MCI, CN vs MCI), and ten-fold cross-validation was employed to ensure result reliability. Evaluation metrics included accuracy (ACC), sensitivity (SEN), specificity (SPEC), area under the receiver operating characteristic curve (AUC), and balanced accuracy (BAC).


(1) $$\begin{array}{l} ACC=\frac{TP+TN}{TP+TN+FP+FN} \end{array}$$

(2) $$\begin{array}{l} SEN=\frac{TP}{TP+FN} \end{array}$$

(3) $$\begin{array}{l} SPEC=\frac{TN}{TN+FP} \end{array}$$

(4) $$\begin{array}{l} BAC=\frac{SEN+SPEC}{2} \end{array}$$

Where TP represents the number of correctly identified positive samples, FP represents the number of negative samples incorrectly classified as positive, FN represents the number of positive samples incorrectly classified as negative, and TN represents the number of correctly identified negative samples.

### 2.3 ADMV-Net framework

**Figure 1:**
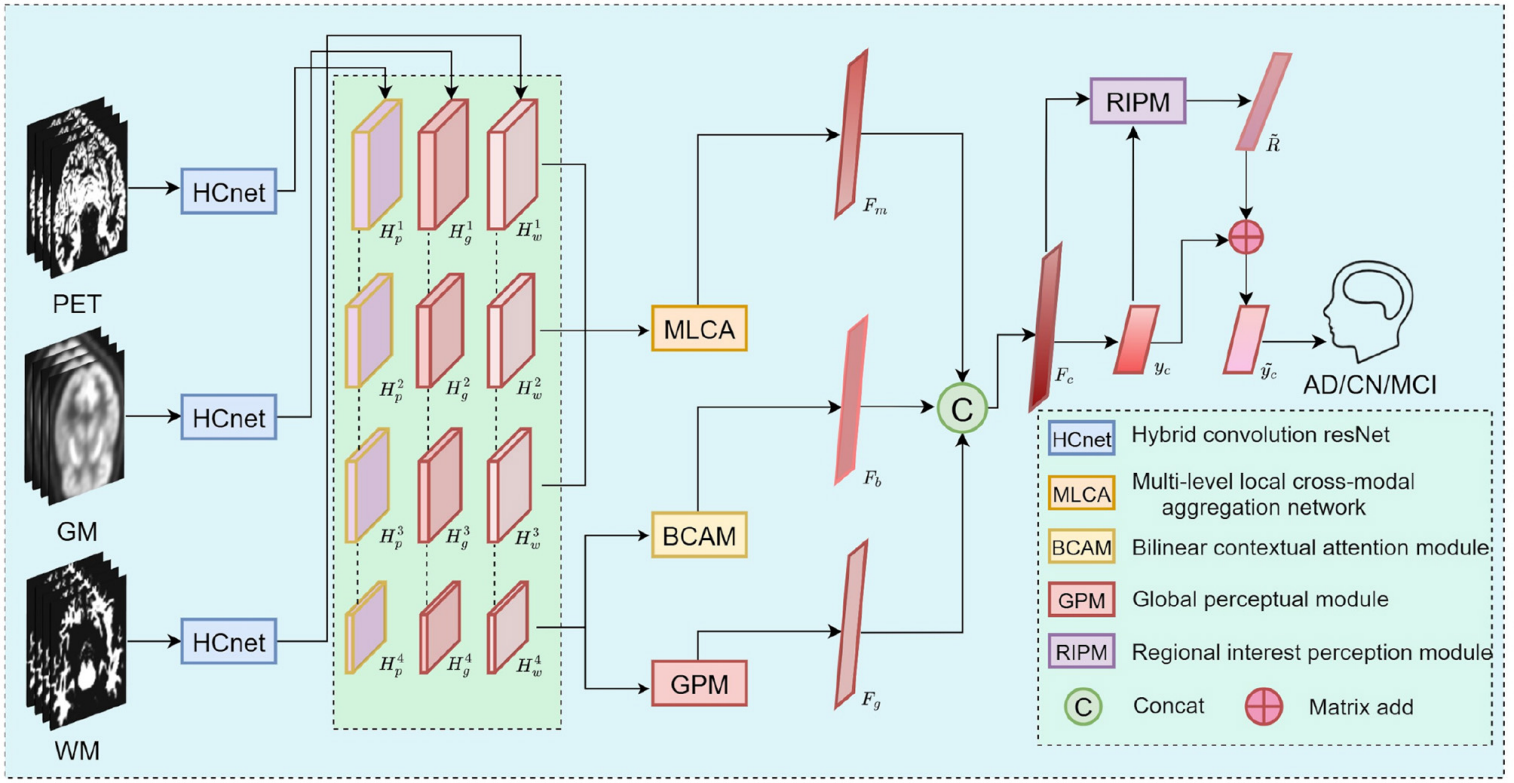
The overall architecture of ADMV-Net. The diagram depicts the primary structural components of the model and the corresponding data processing workflow, illustrating the overall strategy for multimodal feature extraction and fusion.

To address the common challenge of insufficient multimodal fusion in AD diagnosis, this study proposes a multi-view multimodal Alzheimer's disease diagnostic model, ADMV-Net. Existing approaches predominantly rely on simple concatenation or weighted aggregation, which are limited in their ability to simultaneously capture global macroscopic patterns, local key region features, and cross-modal semantic relationships. Moreover, population heterogeneity, label noise, and scanner/site differences further diminish fusion effectiveness and model generalizability. In response, ADMV-Net models inter-modality feature relationships from global, local, and semantic perspectives, incorporating a dynamic brain region weighting mechanism to significantly enhance diagnostic performance. The overall framework is illustrated in Figure 1.

### 2.4 Hybrid convolution ResNet(HCNet)

With the widespread adoption of deep learning in medical image analysis, classic network architectures such as ResNet have become mainstream choices for multimodal feature extraction. However, traditional convolutional networks exhibit notable limitations when handling three-dimensional medical images. On one hand, standard convolutions struggle to capture precise anatomical boundaries. On the other hand, existing network designs fail to effectively balance global semantic information with local structural details. To address these challenges, we propose the Hybrid Convolution ResNet (HCNet). HCNet achieves efficient feature fusion and representation optimization through three parallel pathways.

**Figure 2:**
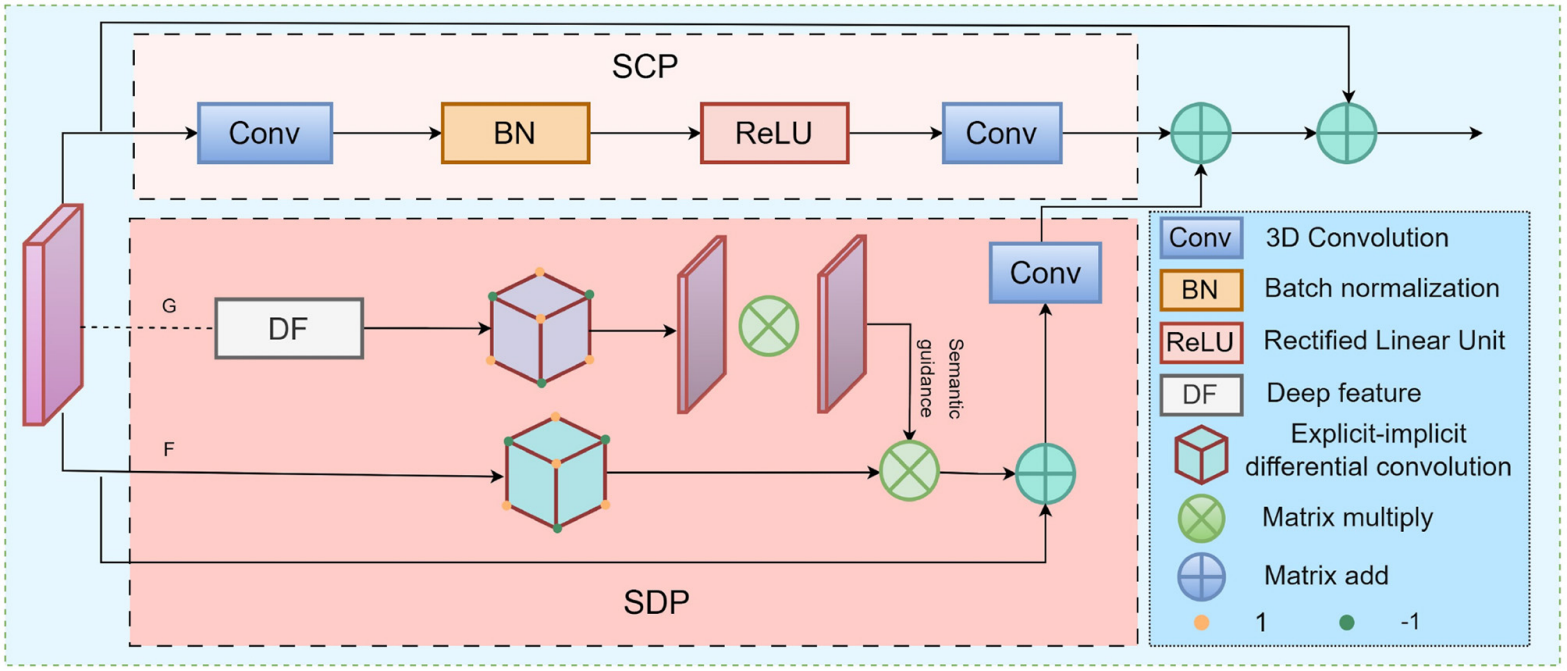
The workflow of HCNet. The dual-path convolutional structure processes global and boundary features separately and fuses them through an adaptive mechanism.

Specifically, the Standard Convolution Path (SCP) builds a large receptive field by stacking deep multi-scale features, indirectly modeling global semantic information; the Semantic Difference-guided Path (SDP) introduces a deep feature map G as a diffusion guide, regulating local feature propagation around boundaries to enhance the perception of ambiguous structural interfaces. The third, a direct mapping path, preserves the integrity of input features to prevent information loss. The detailed architecture is illustrated in Figure 2.

The SCP is based on 3D convolution combined with Batch Normalization and ReLU activation to extract spatial contextual relationships, responsible for capturing macroscopic features and global semantic information. The SDP focuses on overcoming the limitations of standard convolutions in precisely describing boundary regions, accurately characterizing ambiguous interfaces between anatomical structures. Functioning as a “push” mechanism, the SDP reduces boundary uncertainty between classes. This module simulates a nonlinear anisotropic diffusion process, extracting edge features through explicit and implicit differential kernels, while leveraging deep semantic guidance to enhance boundary representation.

Specifically, the iterative update formula of the SCP is given by:


(5) $$\begin{array}{l} {\hat{F}}_{p}^{t+1}=\underset{p_{e}\in \delta_{p}}{\sum }\text{h}\left(||{\text{G}}_{{\text{p}}_{e}}-{\text{G}}_{\text{P}}|{|}^{2}\right)\cdot \left(F_{p_{e}}^{t}-F_{p}^{t}\right) \end{array}$$

(6) $$\begin{array}{l} F^{t+1}=\lambda \cdot F^{t}+\nu \cdot {\hat{F}}^{t+1} \end{array}$$

Where, *F*^*t*^ denotes the feature map at the t iteration, G represents the semantic guidance feature from the deep decoder, h(.) is a learnable nonlinear mapping function, δ_*p*_ denotes the local neighborhood centered at position P, and λ and ν are adjustable weighting parameters.

Finally, the features extracted by SCP and SDP are adaptively fused and combined with those from the direct mapping path, integrating contextual information with boundary-guided cues to enhance the model's representation of ambiguous boundaries.

In ADMV-Net, we employ four HCNet modules to extract feature representations at different hierarchical levels. These multi-level feature maps not only demonstrate the model's capability to progressively capture global semantics and boundary details but also provide a rich foundation of multi-scale representations for subsequent modules. The resulting set of features $\left\{H_{g}^{1},H_{W}^{1},H_{P}^{1},\ldots ,H_{g}^{4},H_{W}^{4},H_{P}^{4}\right\}$ serves as the input to the MVFL module, enabling multi-view fusion modeling.

### 2.5 Global Perception Module

In the task of multimodal Alzheimer's disease diagnosis, modeling global semantic information is crucial for capturing the brain's overall pathological features. However, the spatial resolution loss in deep features extracted by existing models often leads to blurred macroscopic semantic information. Additionally, global correlation patterns across multimodal data are difficult to model effectively through a single pathway. To address these issues, we propose the Global Perception Module (GPM). This module efficiently models global features across multimodal data by leveraging long-range dependency modeling and an adaptive fusion mechanism.

Specifically, we take the output of the last layer of the feature extraction network ,$H_{g}^{4},H_{w}^{4}$ and $H_{P}^{4}$ as the input data. The GPM first applies a 3D convolution for shallow feature extraction, resulting in a feature map F. Next, the key and value of F are fed into a Multi-Head Attention layer, where they interact with F's query to perform attention calculations and obtain enhanced features. Inspired by Yuan et al. (2021)) and Zeng et al. (2022)), we use depthwise separable convolution (DWConv) during feature modeling to capture local features and positional information, while removing explicit positional encoding.

Additionally, after the addition operation, $H_{g}^{4},H_{w}^{4}$ and $H_{P}^{4}$ are fed into the Window Attention module to prevent feature shift between different branches. Here, “feature shift” refers to differences in the distributions of features from different modalities or scales, which can cause one branch to dominate during fusion and thus disrupt overall consistency. The addition operation provides initial alignment, while the sliding-window mechanism of Window Attention adaptively models local interactions, effectively mitigating such shifts and enhancing feature representation with relatively low computational cost.

It is noteworthy that multimodal fusion is influenced not only by biases introduced by confounding factors such as age, sex, scanner manufacturer, and imaging parameters, which can create spurious correlations and reduce model transferability, but also by potential statistical dependencies across different data sources arising from shared biological or pathological mechanisms, measurement procedures, or preprocessing steps. These dependencies further compromise the independence between modalities and the effectiveness of fusion strategies. To mitigate these effects, we introduce RegBN (Ghahremani Boozandani and Wachinger, 2023) prior to feature fusion. RegBN is a regularization-based batch normalization method specifically designed for multimodal data, which removes the need for learnable parameters. This not only simplifies the training and inference pipelines but also helps stabilize feature distributions across modalities.Overall, the data processing workflow of the GPM can be represented as:


(7) $$\begin{array}{l} F_{g}=FFN\left(WA\left(H_{g}^{4}+H_{w}^{4}+H_{P}^{4}\right)H_{g}^{4^{\prime }}H_{w}^{4^{\prime }}H_{p}^{4^{\prime }}\right) \end{array}$$

Where, *WA*(.) denotes the window attention mechanism, while, $H_{g}^{4^{\prime }},H_{w}^{4^{\prime }}$ and $H_{p}^{4^{\prime }}$ represent the features processed through multi-head attention and RegBN, respectively. The processing procedure is described by Equation 8:


(8) $$\{\begin{array}{l} H_{g}^{4^{\prime }}=MHA(H_{g}^{4})\\ H_{w}^{4^{\prime }}=RegBN(H_{g}^{4^{\prime }},MHA(H_{w}^{4}))\\ H_{p}^{4^{\prime }}=RegBN(H_{w}^{4^{\prime }},MHA(H_{p}^{4})) \end{array}$$

**Figure 3:**
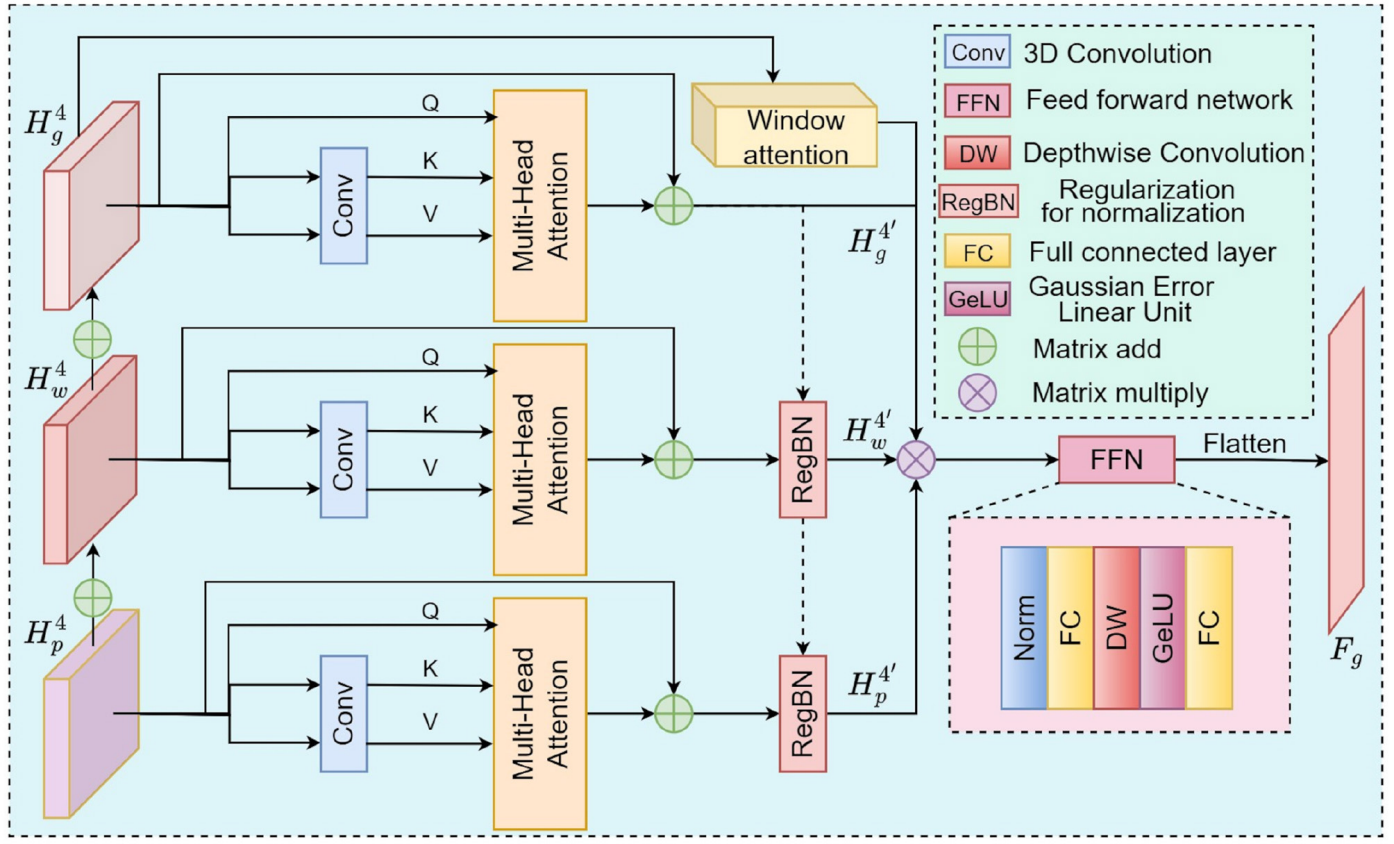
The workflow of GPM. Modeling of multimodal global semantics through multi-head attention and window mechanisms.

Finally, the features fused by the Feed-Forward Network (FFN) are flattened and output as the global fusion features$F_{g}\in {\mathbb{R}}^{c_{4}}$ .The overall architecture is shown in Figure 3.

### 2.6 Multi-level Local Cross-modal Aggregation Module

**Figure 4:**
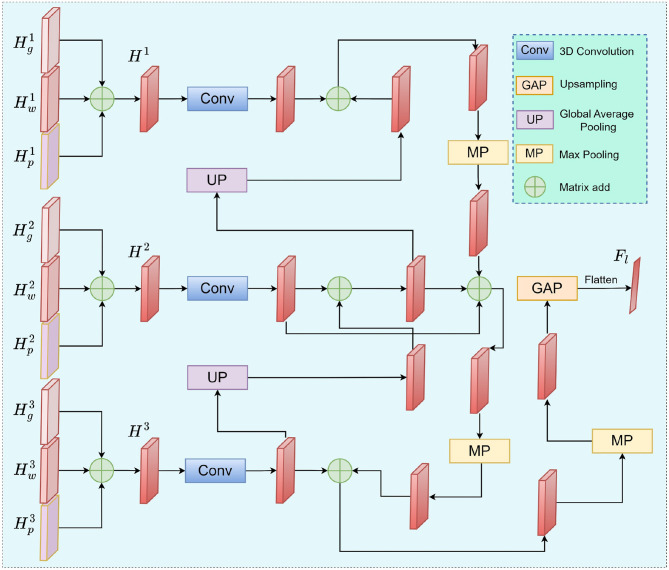
The workflow of MLCA. It fuses local features from different modalities layer by layer and strengthens important feature regions through a weighting mechanism.

In multimodal fusion tasks, MRI and PET modalities exhibit significant complementarity in local detail information. To further enhance the interaction of local information between modalities, we propose the Multi-level Local Cross-modal Aggregation Module (MLCA). This module integrates features from the first three residual blocks to achieve multi-scale semantic fusion. Additionally, it employs a bidirectional pathway and a learnable weighting mechanism to enable deep coupling of local information. The overall architecture is illustrated in Figure 4.

MLCA comprises three main components: (1) Channel alignment, (2) bidirectional feature interaction and reconstruction, and (3) cross-scale fusion and aggregation.

In the Channel alignment stage, the initial fusion features from MRI and PET are denoted as $H^{1},H^{2},H^{3}\in {\mathbb{R}}^{d_{4}\times h_{4}\times w_{4}\times c_{4}}$. These features are projected into a unified 128-channel space via 3D convolutions to eliminate channel dimensional inconsistencies across modalities while preserving the original spatial structures. The aligned feature set is thus expressed as $H^{i}\in {\mathbb{R}}^{d_{4}\times h_{4}\times w_{4}\times 128},i=1,2,3$.

The bidirectional feature interaction and reconstruction stage follows, wherein a dual-path mechanism–comprising top-down and bottom-up pathways–is employed to propagate and refine features across scales. To facilitate cross-scale integration and detailed enhancement, cross-scale weighted fusion nodes are introduced at each level. The output of each fusion node is formulated as:


(9) $$\begin{array}{l} \;F_{out}=\frac{\sum_{i}w_{i}.U\left(F_{i}\right)}{\sum_{i}w_{i}+\epsilon } \end{array}$$

Where, *F*_*i*_ represents the input features from different scales or directions, *W*_*i*_ is the learnable positive weight parameter, *U*(.) denotes the upsampling or downsampling operation, and **ϵ** is the stability factor.

Finally, in the cross-scale aggregation stage, all the fused local features are normalized to a fixed size of 4 × 4 × 4 through global adaptive pooling and then flattened into a one-dimensional vector to form the final local fusion feature $F_{m}\in {\mathbb{R}}^{4}$.

### 2.7 Bilinear Contextual Attention Module (BCAM)

**Figure 5:**
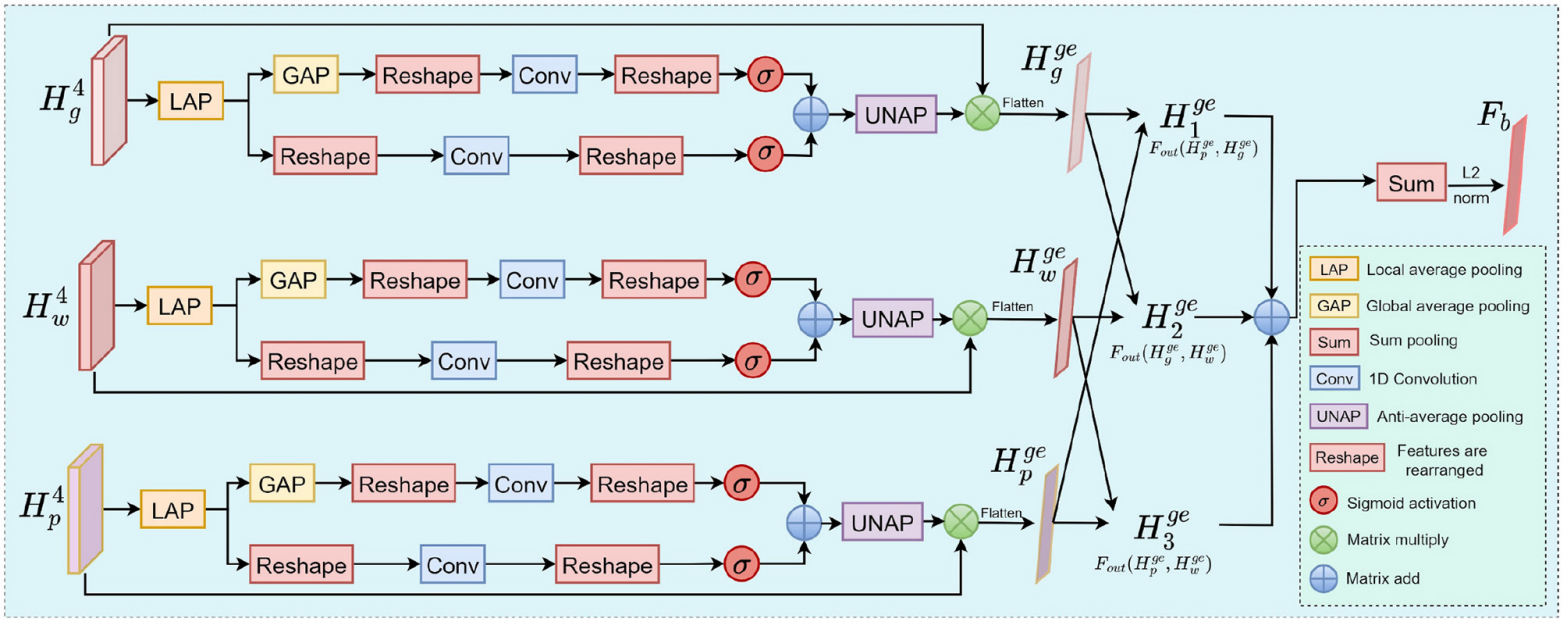
The workflow of BCAM. Latent features are obtained using the outer product operation.

Although existing multimodal feature fusion methods have shown great potential in Alzheimer's disease detection tasks, they often overlook deep interactions between modalities, ignoring latent information. To address this issue, we propose the BCAM to enhance latent feature representation and improve the model's classification performance, as illustrated in Figure 5.

The BCAM begins by performing local and global average pooling on the input features to capture both regional details and overall modality information. After flattening the pooled features, they pass through 1D convolutions for channel dimension compression and weight allocation, thereby implementing a channel-level attention mechanism.

The features are then reshaped to their original dimensions and normalized to the [0,1] interval using the Sigmoid function, producing both local and global attention weights. BCAM employs a dynamic weight fusion strategy to integrate features from various receptive fields. The fused attention map is upsampled to restore its spatial dimensions and then multiplied element-wise with the original features to enhance the key regional information while suppressing redundant features.

After feature enhancement, BCAM introduces a cross-modal interaction mechanism to capture potential correlation patterns between modalities via an outer product operation, thus revealing deep semantic relationships between the modalities.

To reduce computational complexity, we first perform LAP (Local Average Pooling), GAP (Global Average Pooling), and flatten operations on$H_{p}^{4},H_{g}^{4}$ and $H_{w}^{4}$ to obtain the dimensionality-reduced scale features $H_{p}^{ge},H_{g}^{ge},H_{w}^{ge}\in {\mathbb{R}}^{c_{4}\times \frac{d_{a}}{2}}$. Subsequently, by fusing these two sets of features, we acquire the interactive feature representation $H_{1}^{ge},H_{2}^{ge},H_{3}^{ge}\in {\mathbb{R}}^{c_{4}\times \frac{d_{a}}{2}\times \frac{d_{a}}{2}}$.


(10) $$\begin{array}{l} H_{1}^{ge}\left(t\right)=F_{outer}\left(H_{p}^{ge}\left(t\right),H_{g}^{ge}\left(t\right)\right) \end{array}$$

(11) $$\begin{array}{l} H_{2}^{ge}\left(t\right)=F_{outer}\left(H_{g}^{ge}\left(t\right),H_{w}^{ge}\left(t\right)\right) \end{array}$$

(12) $$\begin{array}{l} H_{3}^{ge}\left(t\right)=F_{outer}\left(H_{p}^{ge}\left(t\right),H_{w}^{ge}\left(t\right)\right) \end{array}$$

After the outer product operation, the three sets of fused features are summed. Following this, they are pooled and L2-normalized to obtain the latent feature representation that encapsulates cross-modal correlations.


(13) $$\begin{array}{l} F_{b}=||sumpooling\left(H_{1}^{ge}+H_{2}^{ge}+H_{3}^{ge}\right)|{|}_{2}\in {\mathbb{R}}^{c_{4}} \end{array}$$

Finally, the features *F*_*g*_, *F*_*m*_, and *F*_*b*_ are concatenated and reshaped to form the fused feature representation $F_{c}\in {\mathbb{R}}^{c_{4}}$.

### 2.8 Regional Interest Perception Module (RIPM)

**Figure 6:**
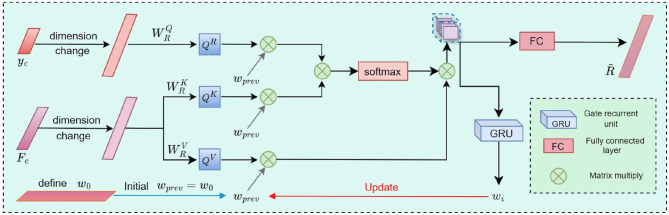
The workflow of RIPM. The module dynamically adjusts the importance weights of different brain regions through t iterations to identify key brain regions.

As a typical stage-progressive neurodegenerative disease, AD exhibits distinct patterns of brain region changes at different stages, which are crucial for early diagnosis and intervention (Qiu et al., 2024). To address this, we employ the Regional Interest Perception Module (RIPM) to identify key brain regions, as shown in Figure 6.

In the implementation, we set the number of iterations to *t* and initialize the brain region weight matrix $\omega_{i}\in {\mathbb{R}}^{1\times 90}$. Through *t* iterations, the importance weights of different brain regions are dynamically adjusted to continuously optimize the weights. In the feature processing stage, the multimodal fusion feature $y_{c}\in {\mathbb{R}}^{2}$ is first dimensionally transformed to generate multimodal classification features and initial Region of Interest (ROI) features $R_{0}\in {\mathbb{R}}^{1\times 90}$. Subsequently, *y*_*c*_ is feature-transformed to generate the hidden feature ${ŷ}_{c}^{R}\in {\mathbb{R}}^{1\times 90}$.

To deeply capture the interaction information between ROIs, we use the query weight matrix $W_{R}^{Q}\in {\mathbb{R}}^{90\times 90}$, key weight matrix $W_{R}^{K}\in {\mathbb{R}}^{90\times 90}$, and value weight matrix $W_{R}^{V}\in {\mathbb{R}}^{90\times 90}$. By performing linear mapping with ${ỹ}_{c}^{R}$ and *R*_0_, we obtain ${\text{Q}}^{R}\in {\mathbb{R}}^{c_{4}\times 90}$, $K^{R}\in {\mathbb{R}}^{c_{4}\times 90}$, $V^{R}\in {\mathbb{R}}^{c_{4}\times 90}$, calculated as follows:


(14) $$\begin{array}{l} Q^{R}=W_{R}^{Q}{ỹ}_{c}^{R},K^{R}=W_{R}^{K}R_{0},V^{R}=W_{R}^{V}R_{0} \end{array}$$

Subsequently, the ROI weight matrix is continuously updated using a Gated Recurrent Unit (GRU).


(15) $$\begin{array}{l} {\text{R}}_{h}=\left(softmax\left(\left(\omega_{prev}{\text{Q}}^{R}\right){\left(\omega_{prev}{\text{K}}^{R}\right)}^{T}\right)\right)*\omega_{prev}{\text{V}}^{R} \end{array}$$

(16) $$\begin{array}{l} \omega_{i}=F_{GRU}\left({\text{R}}_{h}^{T},w_{prev}^{T}\right) \end{array}$$

After *t* rounds of iterative updates, the final salient brain region feature $R_{h}\in {\mathbb{R}}^{1\times 90}$ is obtained. *R*_*h*_ is linearly mapped to generate the final ROI feature $\tilde{R}\in {\mathbb{R}}^{90}$. Further, $\tilde{R}$ is dimensionally transformed to obtain $y_{c}^{r}\in {\mathbb{R}}^{2}$, which is then weighted and fused with the multimodal classification feature *y*_*c*_ to form the final classification feature ${ŷ}_{c}\in {\mathbb{R}}^{2}$. By dynamically adjusting the importance weights of different brain regions, this approach provides a more effective research strategy for salient brain region extraction and multimodal feature optimization.

## 3 Results

### 3.1 Optimal iteration number analysis

To determine the optimal interpretability parameters of ADMV-Net across the three classification tasks, we systematically evaluated the effect of the number of iterations in the RIPM module on both model performance and the stability of brain region weights, with the results presented in Table 1. From a quantitative interpretability perspective, the AD vs CN and MCI vs CN tasks achieved peak performance at seven iterations, indicating that a moderate number of iterations allows RIPM to effectively capture inter-regional brain interaction patterns while maintaining biologically plausible weight distributions. By comparison, the AD vs MCI task indicates that excessive iterations may introduce noise features unrelated to disease pathology, thereby reducing the biological interpretability of the model. Based on these quantitative analyses, we employed the corresponding optimal number of iterations for each task, ensuring that the RIPM module provides stable and reliable weight assignments for each brain region, thus offering clinicians quantitative insights into region-specific importance.

**Table 1:** Performance comparison across different tasks and datasets.

**Task**	**ADNI**					**AIBL**				
	**1**	**3**	**5**	**7**	**9**	**1**	**3**	**5**	**7**	**9**
AD vs. CN	94.52 ± 3.83	93.87 ± 3.26	94.05 ± 5.48	**94.83 ± 2.76**	93.64 ± 2.76	94.85 ± 4.33	93.46 ± 2.66	94.37 ± 3.62	**95.26 ± 3.84**	93.35 ± 5.27
MCI vs. CN	72.15 ± 4.34	71.19 ± 3.58	71.84 ± 3.49	**72.71 ± 3.17**	71.77 ± 3.17	78.38 ± 5.86	77.95 ± 4.23	77.64 ± 5.51	**78.64 ± 8.62**	77.51 ± 4.43
AD vs. MCI	**85.91 ± 3.24**	83.96 ± 5.47	83.35 ± 4.43	85.66 ± 4.47	83.17 ± 3.82	**89.27 ± 4.31**	88.45 ± 5.74	88.72 ± 3.36	88.95 ± 6.24	87.82 ± 4.25

Best results are bolded.

### 3.2 Multi-fold loss curve analysis

**Figure 7:**
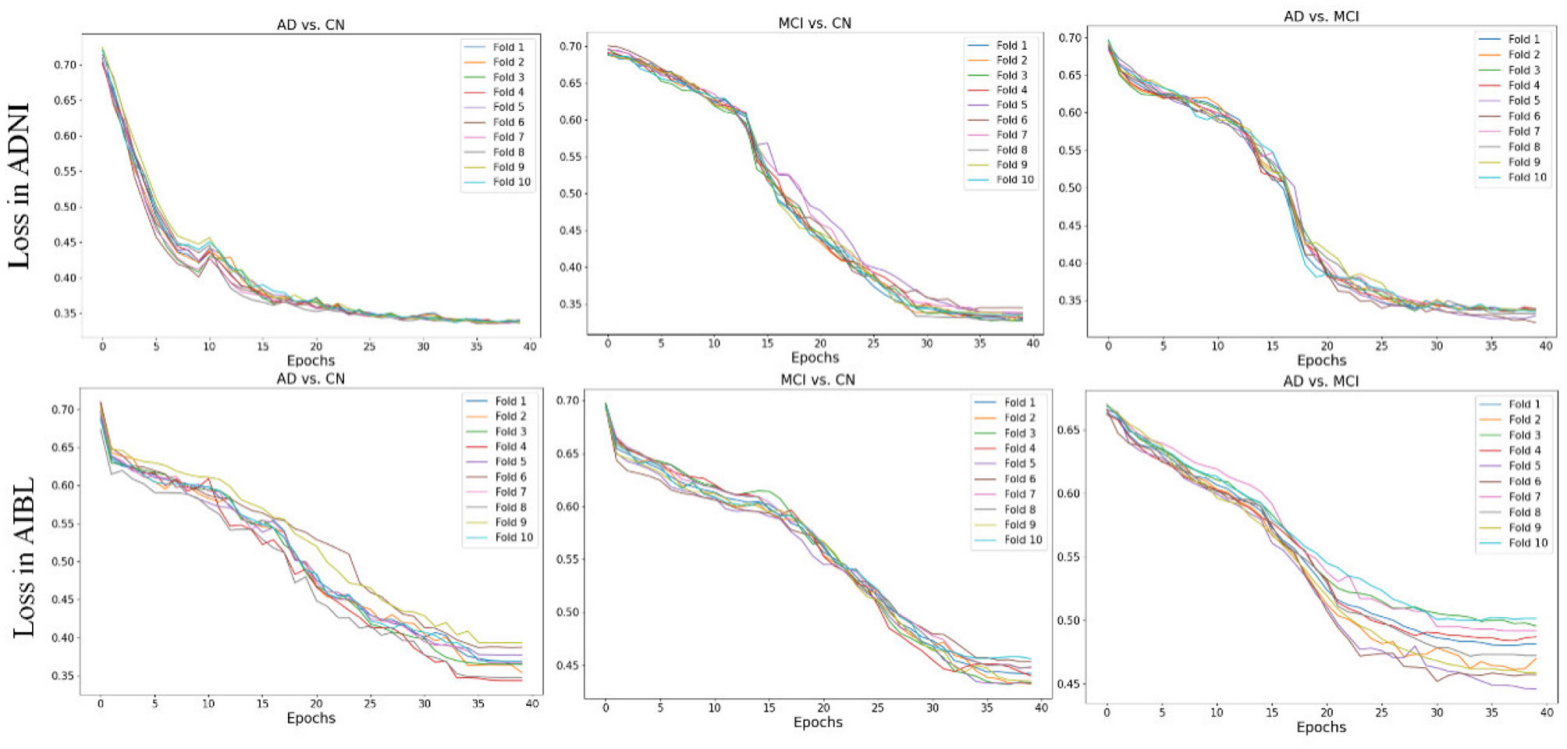
Loss Curves for different tasks.

Through analysis of loss curves from multi-fold cross-validation, as shown in Figure 7, we found that the loss change trends across different folds remain consistent on both the large-scale ADNI dataset and the smaller AIBL dataset, strongly validating the stability of the method and reliability of the results.

### 3.3 Ablation experiment results

#### 3.3.1 Feature extraction network ablation

**Figure 8:**
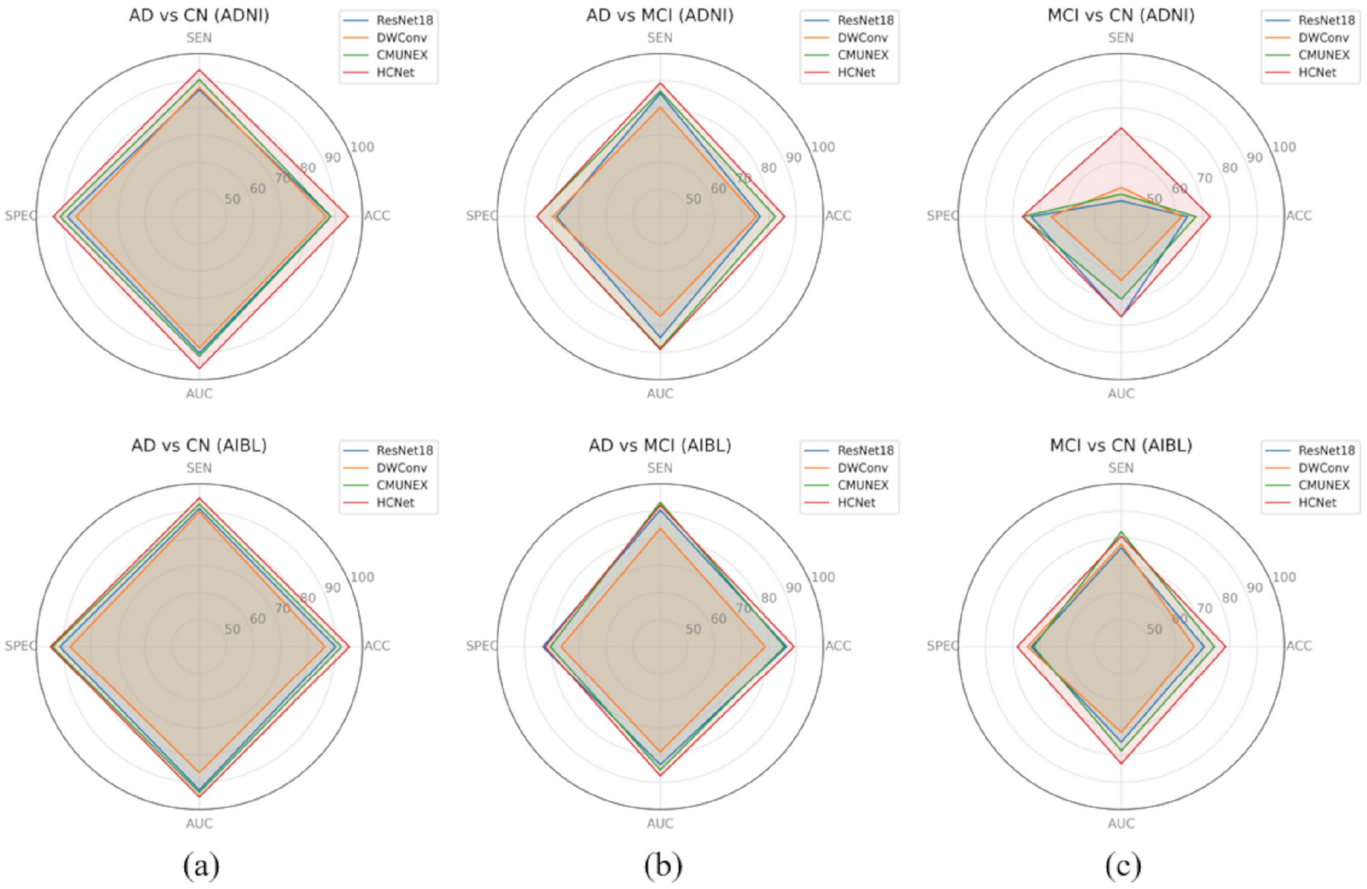
Radar charts illustrating the performance of HCNet and other comparative models on different classification tasks across the ADNI and AIBL datasets. **(a)** AD vs CN task; **(b)** AD vs MCI task; **(c)** MCI vs CN task.

To comprehensively evaluate HCNet's performance in 3D medical image feature extraction, we conducted performance tests for three classification tasks on both ADNI and AIBL datasets. Results in Table 2 show that HCNet achieved optimal performance in AD vs CN tasks across both datasets. In the more challenging MCI vs CN and AD vs MCI tasks, HCNet achieved the highest accuracy (72.77% and 85.81%, respectively) and sensitivity (72.69% and 89.27%, respectively), demonstrating good generalization capability. Although its specificity was slightly lower than CMUNEX, HCNet still maintained significant advantages in overall performance. In contrast, ResNet18 and DWConv showed relatively unstable performance, further highlighting HCNet's advantages in identifying MCI patients. The statistical test results indicate that the improvements of HCNet in the main performance indicators are significant. To more intuitively demonstrate the comprehensive performance of each model across evaluation metrics, we created the radar chart shown in Figure 8. The area size in the chart reflects the overall performance of the model across four metrics: ACC, SEN, SPEC, and AUC, with larger areas indicating better model performance.

**Table 2:** Performance comparison of different feature extraction networks on the three classification tasks.

**Task**		**ADNI**				**AIBL**			
		**ResNet18**	**DWConv**	**CMUNEX**	**HCNet**	**ResNet18**	**DWConv**	**CMUNEX**	**HCNet**
AD vs. CN	ACC	88.34 ± 6.42	86.7 ± 7.29	88.5 ± 4.83	**94.83** **±2.76**	90.2 ± 1.3.25	86.3 ± 5.64	92.33 ± 3.65	**95.26** **±3.84**
	SEN	86.72 ± 7.41	87.54 ± 7.26	90.43 ± 7.63	**94.07** **±4.95**	90.87 ± 4.53	89.64 ± 6.21	92.58 ± 4.29	**94.74** **±5.66**
	SPEC	88.65 ± 4.63	85.44 ± 8.58	91.32 ± 3.07	**93.76** **±4.24**	91.34 ± 6.37	87.56 ± 5.28	94.07 ± 2.58	**94.81** **±2.97**
	AUC	90.27 ± 5.62	88.31 ± 6.34	91.42 ± 4.34	**95.97** **±2.63**	92.84 ± 3.65	86.49 ± 6.26	93.78 ± 5.24	**95.43** **±4.54**
	*p*-value	*p* < 0.001	*p* < 0.001	*p* < 0.001	-	*p* < 0.001	*p* < 0.001	*p* < 0.001	-
MCI vs. CN	ACC	64.37 ± 4.34	62.29 ± 4.85	67.52 ± 8.62	**72.77** **±3.17**	70.51 ± 6.74	66.85 ± 10.37	74.33 ± 3.66	**78.46** **±8.62**
	SEN	45.79 ± 15.43	50.74 ± 8.86	48.24 ± 17.62	**72.69** **±5.34**	76.43 ± 5.42	77.79 ± 6.44	**82.35** **±4.26**	80.73 ± 4.19
	SPEC	73.24 ± 5.26	65.87 ± 6.52	**76.49** **±9.24**	76.41 ± 7.53	73.18 ± 4.33	74.56 ± 9.26	72.33 ± 6.37	**78.34** **±6.75**
	AUC	**76.93** **±2.77**	63.48 ± 5.42	70.39 ± 6.17	76.84 ± 3.92	75.26 ± 5.56	71.67 ± 7.27	78.45 ± 5.11	**83.21** **±6.44**
	*p*-value	*p* < 0.001	*p* < 0.001	*p* < 0.001	-	*p* < 0.001	*p* < 0.001	*p* < 0.001	-
AD vs. MCI	ACC	76.82 ± 2.77	75.46 ± 11.52	82.34 ± 7.68	**85.81** **±3.24**	86.52 ± 4.39	78.66 ± 14.31	85.74 ± 5.79	**89.27** **±4.31**
	SEN	85.37 ± 4.68	80.21 ± 9.75	86.25 ± 4.37	**89.27** **±4.86**	90.38 ± 4.22	83.61 ± 9.87	**93.24** **±2.88**	92.16 ± 3.62
	SPEC	78.16 ± 9.29	79.57 ± 12.11	**85.53** **±7.25**	85.46 ± 3.79	**83.25** **±3.26**	76.54 ± 9.15	80.46 ± 2.17	82.4 ± 9.15
	AUC	84.56 ± 6.64	76.78 ± 9.82	88.49 ± 6.44	**88.93** **±3.42**	83.59 ± 6.42	78.89 ± 7.16	85.48 ± 5.62	**87.66** **±4.39**
	*p*-value	*p* < 0.001	*p* < 0.001	*p* < 0.001	-	*p* < 0.001	*p* < 0.001	*p* < 0.001	-

Best results are bolded.

#### 3.3.2 Module ablation

To validate the effectiveness of each component in ADMV-Net, we conducted systematic ablation experiments, with the results presented in Table 3. Analysis of the individual contributions of each module indicates that the GPM module, through its multi-head self-attention mechanism, establishes long-range dependencies. Combined with the brain region weighting of RIPM, it achieved 92.43% ACC and 94.57% AUC in the AD vs CN task, demonstrating the foundational role of global semantic awareness. The MLCA module, leveraging bidirectional feature interaction for deep cross-scale local information aggregation, further enhanced performance when integrated with RIPM, reaching 93.25% ACC and 94.68% AUC. The BCAM module, which explores latent inter-modality associations via outer product operations, achieved 91.95% ACC with RIPM alone, but its deep semantic modeling capabilities became evident in subsequent module combinations.

**Table 3:** Performance comparison of ablation results in the three classification tasks.

**Task**	**GPM**	**MLCA**	**BCAM**	**RIPM**	**ACC**	**SEN**	**SPEC**	**AUC**	***p*-value**
	✓			✓	92.43 ± 2.62	92.74 ± 3.32	91.71 ± 3.92	94.57 ± 2.48	*p* < 0.001
		✓		✓	93.25 ± 2.81	92.62 ± 2.15	93.85 ± 3.98	94.68 ± 1.86	*p* < 0.001
			✓	✓	91.95 ± 4.34	92.73 ± 3.58	92.74 ± 3.66	93.88 ± 3.72	*p* < 0.001
AD vs. CN	✓	✓		✓	94.73 ± 4.36	**95.56** **±5.24**	**95.11** **±2.84**	94.95 ± 3.62	*p* < 0.05
	✓		✓	✓	93.86 ± 8.34	94.08 ± 6.56	94.15 ± 7.59	94.66 ± 5.81	*p* < 0.001
		✓	✓	✓	92.04 ± 7.62	93.93 ± 9.27	93.65 ± 6.24	94.72 ± 6.34	*p* < 0.001
	✓	✓	✓		93.59 ± 4.78	93.96 ± 3.61	93.55 ± 3.17	94.22 ± 2.39	*p* < 0.001
	✓	✓	✓	✓	**94.83** **±2.76**	94.07 ± 4.95	93.76 ± 4.24	**95.97** **±2.62**	-
	✓			✓	70.94 ± 3.04	70.53 ± 4.32	75.43 ± 5.62	74.26 ± 3.83	*p* < 0.001
		✓		✓	71.26 ± 2.78	72.01 ± 3.64	76.31 ± 3.87	75.88 ± 4.52	*p* < 0.05
			✓	✓	70.65 ± 4.82	70.16 ± 7.63	75.02 ± 8.34	74.22 ± 7.61	*p* < 0.001
MCI vs CN	✓	✓		✓	72.09 ± 3.16	71.74 ± 2.89	76.54 ± 3.42	76.34 ± 5.58	*p* < 0.001
	✓		✓	✓	71.34 ± 5.24	71.56 ± 6.17	75.73 ± 6.35	75.70 ± 6.42	*p* < 0.001
		✓	✓	✓	71.33 ± 4.51	70.95 ± 7.33	**76.73** **±6.35**	75.73 ± 6.42	*p* < 0.01
	✓	✓	✓		71.48 ± 3.02	71.86 ± 5.36	76.34 ± 5.47	75.25 ± 3.64	*p* < 0.001
	✓	✓	✓	✓	**72.77** **±3.17**	**72.69** **±5.34**	76.41 ± 7.53	**76.84** **±3.92**	-
	✓			✓	81.64 ± 4.67	85.93 ± 6.22	82.48 ± 5.32	86.39 ± 4.02	*p* < 0.001
		✓		✓	83.70 ± 3.95	84.76 ± 11.74	83.73 ± 4.55	87.56 ± 5.64	*p* < 0.01
			✓	✓	81.48 ± 8.82	85.01 ± 8.61	83.29 ± 7.73	87.13 ± 2.83	*p* < 0.05
AD vs MCI	✓	✓		✓	83.95 ± 5.67	86.22 ± 4.37	83.56 ± 10.95	86.74 ± 5.58	*p* < 0.001
	✓		✓	✓	82.32 ± 6.73	**89.36** **±5.94**	84.16 ± 4.58	88.42 ± 2.85	*p* < 0.001
		✓	✓	✓	84.25 ± 7.62	87.45 ± 7.42	83.82 ± 6.74	87.94 ± 5.62	*p* < 0.01
	✓	✓	✓		83.58 ± 2.89	87.26 ± 6.43	84.07 ± 3.85	86.27 ± 4.16	*p* < 0.001
	✓	✓	✓	✓	**85.81** **±3.24**	89.27 ± 4.86	**84.66** **±3.79**	**88.93** **±3.42**	-

Best results are bolded.

The synergistic effects between modules were more pronounced. The combined configuration of GPM and MLCA (GPM+MLCA+RIPM) increased performance to 94.73% ACC and 94.95% AUC, with SEN reaching 95.56%, fully demonstrating the complementarity between global and local features. Similarly, the combination of GPM and BCAM (GPM+BCAM+RIPM) achieved 93.86% ACC, highlighting the effective integration of global awareness and latent feature learning. Critical ablation experiments showed that removing RIPM from the three core modules (GPM+MLCA+BCAM) led to a decrease in performance to 93.59% ACC and 94.22% AUC, underscoring the key role of brain region weighting.

When the complete model integrated all four components, it achieved optimal performance across all tasks: 94.83% ACC and 95.97% AUC for AD vs CN, 72.77% ACC and 76.84% AUC for MCI vs CN, and 85.81% ACC and 88.93% AUC for AD vs MCI. These results fully validate the effectiveness of the multi-view fusion architecture and the complementary synergistic contributions of its components.

### 3.4 Comparison experiment

#### 3.4.1 Cross-dataset validation

**Figure 9:**
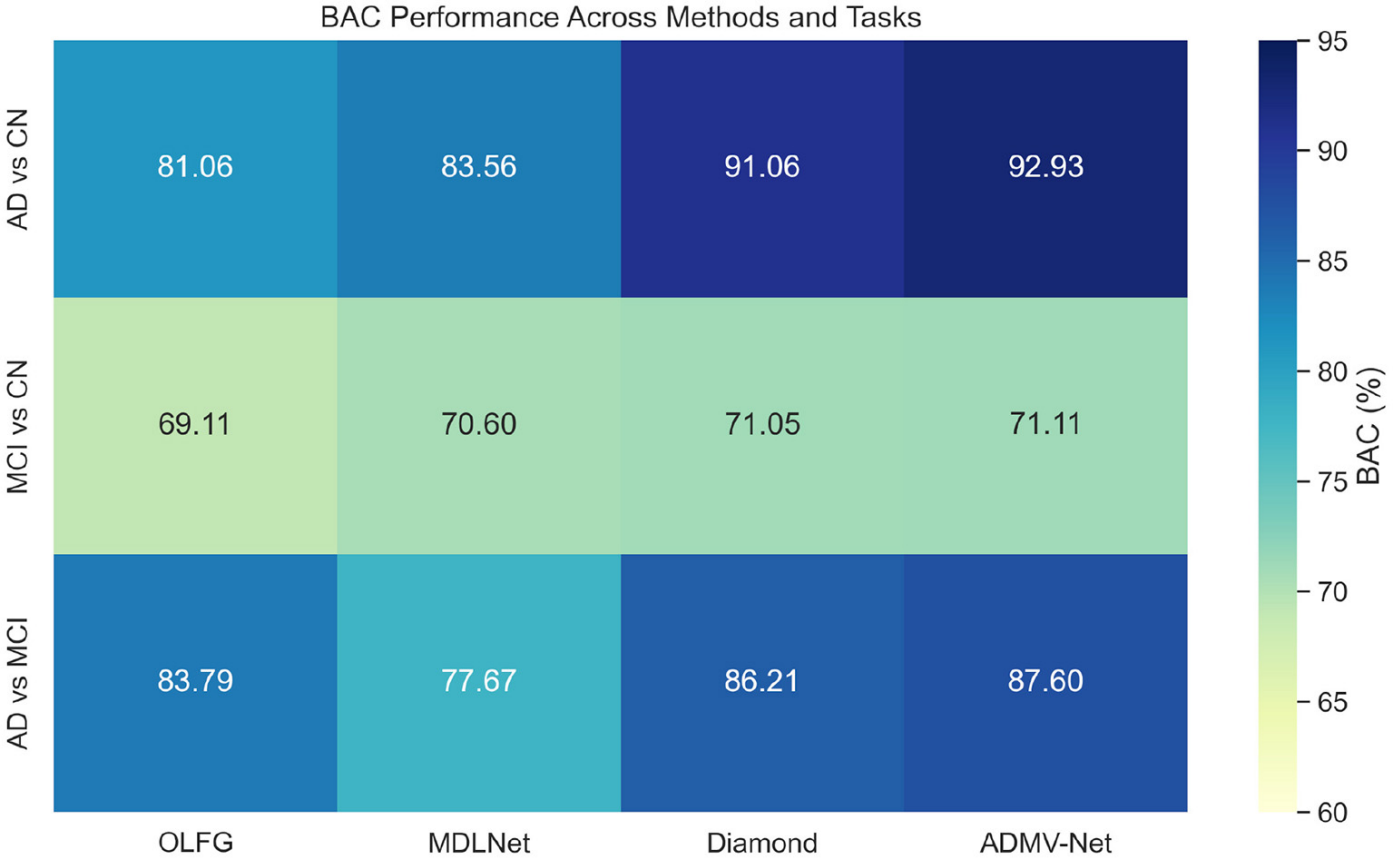
Heatmaps of BAC scores for different models in cross-dataset validation. The closer the color is to blue-green, the better the model's performance.

To validate the cross-dataset generalization capability of ADMV-Net, we designed rigorous cross-validation experiments. After training the model on the ADNI dataset, we tested it on the AIBL dataset to evaluate the model's adaptability and stability across different datasets. To balance potential bias between sensitivity (SEN) and specificity (SPEC) caused by different data distributions, we introduced balanced accuracy (BAC) as a core evaluation metric. Experimental results shown in Table 4 demonstrate that ADMV-Net not only achieved the highest accuracy (ACC) and area under the curve (AUC) across all three tasks (92.37%/94.51%, 71.66%/74.86%, and 90.78%/88.07%, respectively), but also consistently outperformed other comparative methods on the BAC metric, fully demonstrating its strong discriminative capability and consistency across different cognitive impairment classification tasks. A more intuitive representation is shown in Figure 9, which displays a heatmap of BAC for different models, where colors closer to blue-green indicate better performance.

**Table 4:** Cross-dataset validation results.

**Task**	**Method**	**ACC**	**SEN**	**SPEC**	**BAC**	**AUC**	***p*-value**
	OLFG	80.35	78.62	83.49	81.06	86.83	p < 0.001
AD vs CN	MDLNet	78.46	83.75	83.37	83.56	85.69	*p* < 0.001
	Diamond	86.74	89.53	**92.58**	91.06	93.26	*p* < 0.001
	ADMV-Net	**92.37**	**93.44**	92.41	**92.93**	**94.51**	-
	OLFG	62.87	**72.67**	65.34	69.11	69.74	*p* < 0.001
MCI vs CN	MDLNet	56.39	68.58	**73.64**	70.60	68.58	*p* < 0.001
	Diamond	70.28	71.82	70.28	71.05	71.42	*p* < 0.001
	ADMV-Net	**71.66**	72.14	69.05	**71.11**	**74.86**	-
	OLFG	78.43	85.25	82.32	83.79	79.77	*p* < 0.001
AD vs MCI	MDLNet	79.05	76.91	78.43	77.67	74.68	*p* < 0.001
	Diamond	87.72	**91.63**	80.79	86.21	86.12	*p* < 0.001
	ADMV-Net	**90.78**	91.59	**83.61**	**87.60**	**88.07**	-

ADNI as the training set and AIBL as the test set (best results are bolded).

#### 3.4.2 Model comparison

To comprehensively evaluate the effectiveness of the proposed method, we conducted a systematic comparison with state-of-the-art approaches across three classification tasks. The experimental results are presented in Table 5. In the AD vs CN task, ADMV-Net achieved an accuracy (ACC) of 94.83% and an area under the ROC curve (AUC) of 95.97%, outperforming the current best-performing model, Diamond, which attained 92.37% and 94.53%, respectively. Furthermore, sensitivity (SEN) and specificity (SPEC) reached 94.67% and 93.76%, respectively, indicating that the model not only improves overall classification accuracy but also maintains a low false-positive rate.

**Table 5:** Performance comparison with state-of-the-art methods.

**Task**	**Method**	**ACC**	**SEN**	**SPEC**	**AUC**	**#Param(M)**	**FLOPs(G)**	***p*-value**
	OLFG	86.24 ± 6.47	81.98 ± 9.64	89.93 ± 7.64	90.28 ± 4.46	34.25	135.47	*p* < 0.001
AD vs CN	MDLNet	88.42 ± 3.59	85.69 ± 4.15	89.73 ± 3.53	92.64 ± 3.12	**10.97**	21.43	*p* < 0.001
	Diamond	92.37 ± 3.27	94.58 ± 2.61	**94.04** **±4.22**	93.53 ± 3.24	24.53	97.62	*p* < 0.001
	ADMV-Net	**94.83** **±2.76**	**94.67** **±4.95**	93.76 ± 4.24	**95.97** **±2.63**	11.04	**18.95**	-
	OLFG	68.59 ± 4.72	55.38 ± 10.53	68.02 ± 9.35	70.94 ± 6.77	34.25	134.52	*p* < 0.001
MCI vs CN	MDLNet	70.64 ± 4.51	**74.25** **±6.37**	74.88 ± 5.79	75.36 ± 5.78	**10.96**	21.73	*p* < 0.001
	Diamond	70.56 ± 5.43	70.11 ± 6.59	73.45 ± 5.14	73.89 ± 6.73	24.54	98.65	*p* < 0.001
	ADMV-Net	**72.77** **±3.17**	72.69 ± 5.34	**76.41** **±7.53**	**76.84** **±3.94**	11.02	**19.44**	-
	OLFG	81.29 ± 4.66	81.12 ± 4.47	82.16 ± 5.03	84.34 ± 3.94	34.31	135.78	*p* < 0.001
AD vs MCI	MDLNet	81.36 ± 7.35	83.56 ± 6.28	**84.73** **±6.75**	82.39 ± 6.57	**10.96**	22.55	*p* < 0.001
	Diamond	80.91 ± 3.83	**90.64** **±4.74**	82.26 ± 5.18	82.17 ± 4.86	24.57	97.73	*p* < 0.001
	ADMV-Net	**85.81** **±3.24**	89.27 ± 4.86	84.56 ± 3.79	**88.93** **±3.42**	11.04	**18.82**	-

Best results are bolded.

**Figure 10:**
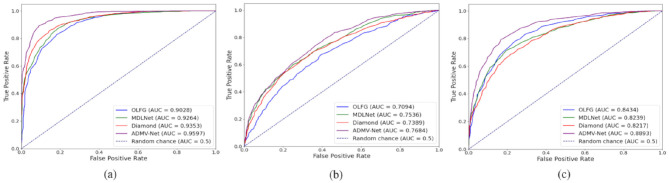
ROC curves of different models for the three classification tasks. **(a)** AD vs CN task; **(b)** MCI vs CN task; **(c)** AD vs MCI task.

In terms of computational efficiency, ADMV-Net also demonstrated substantial advantages. The model comprises only 11.04 million parameters, markedly fewer than OLFG's 34.25M and Diamond's 24.53M, while requiring 18.95 GFLOPs, far lower than OLFG's 133.47 GFLOPs. This computational efficiency enhances the feasibility of deploying ADMV-Net on standard clinical hardware, enabling support for real-time clinical decision-making. For visual illustration, Figure 10 presents the ROC curves of the models, where a larger area under the curve indicates superior performance.

In the more challenging MCI vs CN task, ADMV-Net continued to exhibit strong discriminative capability and stability, achieving an ACC of 72.77%, AUC of 76.84%, and SPEC of 76.41%, thereby validating its effectiveness for early screening of mild cognitive impairment. In the AD vs MCI task, the model maintained leading performance with ACC and AUC values of 85.81% and 88.93%, respectively, and achieved a favourable balance between SEN (89.27%) and SPEC (84.56%), further demonstrating its robust performance in distinguishing different stages of cognitive impairment and highlighting its potential for clinical application.

## 4 Discussion

### 4.1 Model performance and core advantages

Based on the comprehensive experimental results from Tables 2–5, ADMV-Net demonstrates significant performance advantages in multimodal Alzheimer's disease diagnostic tasks. These advantages are primarily reflected in the following three synergistic improvements.

In feature extraction, traditional 3D convolution methods (Kim et al., 2024; Gao et al., 2023; Pandey et al., 2024) struggle to balance global semantic information with local details in medical image processing. To address this issue, we propose a dual-pathway convolution structure, HCNet. This structure captures global semantic and local edge information through parallel channels, effectively resolving this contradiction and significantly improving the accuracy and completeness of feature representation.

Regarding multimodal fusion strategies, existing research primarily employs simple feature concatenation or weighted averaging methods (Lin et al., 2017; Liu et al., 2023a; Bravo-Ortiz et al., 2024), which fail to fully exploit deep complementary information between different modalities. To address this limitation, we designed the MVFL mechanism, which performs interactive fusion of sMRI and PET features from three views: global (GPM), local (MLCA), and latent association (BCAM). Ablation experiments validated the effectiveness of MVFL in consistently improving model performance and enhancing fine-grained cognitive difference capture capabilities, demonstrating the advantages of multi-view fusion.

Furthermore, to enhance model interpretability, we utilize RIPM to automatically learn brain region weight matrices through a data-driven approach, highlighting key brain regions associated with disease and reducing dependence on traditional population-based statistical region-of-interest methods (Kwon, 2023; Qiao et al., 2022; Cao et al., 2017).

### 4.2 Performance comparison and method evaluation

Compared to current mainstream methods, ADMV-Net demonstrates significant advantages across all tasks. Specifically, in the AD vs CN task, our model improves accuracy from Diamond's (Li et al., 2024) 92.37% to 94.83%, while AUC also increases from 94.53% to 95.97%. In the more challenging MCI vs CN task, ADMV-Net continues to outperform methods such as OLFG and MDLNet, fully demonstrating the sensitivity of the multi-view fusion strategy to subtle cognitive differences. More importantly, cross-dataset validation shows that under the ADNI training and AIBL testing setup, ADMV-Net maintains leading performance, indicating good adaptability to changes in data distribution. Notably, some studies (Chen et al., 2025; Zhang et al., 2023) indicate that validation on only a single dataset without cross-dataset validation fails to comprehensively assess model generalization capability, thereby limiting application potential in real-world scenarios. In contrast, the cross-dataset validation employed in this paper further highlights ADMV-Net's advantages in model robustness and practicality.By analyzing the model's misclassification cases, we observed that the primary failure modes of ADMV-Net are concentrated around borderline cases. In the AD vs MCI task, misclassifications predominantly occurred for early-stage AD patients (misidentified as MCI) and late-stage MCI patients (misidentified as AD). In the MCI vs CN task, errors were mainly associated with the identification of mild MCI patients, aligning closely with the known challenges in clinical diagnosis. These findings underscore the inherent difficulty of early Alzheimer's disease detection and provide valuable guidance for future model refinement.

### 4.3 Clinical implications

ADMV-Net demonstrates considerable potential for clinical application. By leveraging both sMRI and PET data, the method can significantly enhance the accuracy of early Alzheimer's disease diagnosis, particularly in distinguishing AD patients from cognitively normal individuals (CN). This capability enables clinicians to identify high-risk individuals at an earlier stage, allowing timely intervention and potentially improving patient prognosis. Moreover, the RIPM module further highlights the importance of specific brain regions, providing clinicians with a clearer understanding of the model's decision-making rationale, thereby increasing confidence in diagnostic outcomes and supporting informed clinical decision-making. Collectively, these advantages suggest that ADMV-Net is not only suitable for early diagnosis but can also assist in long-term disease monitoring, tracking disease progression, and evaluating treatment efficacy, offering a more comprehensive resource for clinical management.

### 4.4 Research limitations and future prospects

While this study has achieved promising results, several limitations remain that warrant further improvement. First, the analysis was based solely on sMRI and PET modalities, a choice primarily motivated by data availability and methodological comparability. Nonetheless, other imaging modalities, such as fMRI and DTI, offer valuable insights into functional network dynamics and white matter structural connectivity, which are also critical for the early detection of Alzheimer's disease. Future work will aim to extend multimodal integration and multi-omics fusion to achieve a more precise and comprehensive modelling of disease mechanisms.

Second, the current validation relied exclusively on the publicly available ADNI and AIBL datasets. Although these datasets are of high quality and well-standardized, they may not fully capture the complexity of real-world clinical settings. In future studies, we plan to conduct multicentre clinical validation, incorporating both retrospective analyses and prospective studies to evaluate the model's performance in practical diagnostic workflows, thereby enhancing the robustness and clinical applicability of ADMV-Net.

Finally, this study employed cross-sectional data analysis, focusing on the static differentiation of distinct cognitive states. While effective in distinguishing AD, MCI, and CN conditions, it lacks dynamic modelling of disease progression. Future research will integrate longitudinal data to track multimodal imaging changes over time, enabling the development of predictive models for disease progression and providing guidance on optimal timing for clinical interventions.

## 5 Conclusion

The ADMV-Net model proposed in this study demonstrates excellent performance in multimodal Alzheimer's disease diagnostic tasks, effectively fusing complementary information from MRI and PET while enhancing feature representation through multi-view mechanisms and accurately identifying key brain regions. Experimental results show that ADMV-Net outperforms existing advanced methods across multiple classification tasks, achieving 94.83% accuracy and 95.97% AUC in AD versus CN classification tasks, with good generalization capability and robustness. This model not only achieves important technical breakthroughs and proposes innovative solutions in multi-view fusion and feature extraction, but also provides strong technical support for early AD diagnosis and clinical applications. In the future, we will continue to deepen related research and promote further development and application of multimodal deep learning in the field of neurodegenerative disease diagnosis.

## Data Availability

Publicly available datasets were analyzed in this study. This data can be found here: This study analyzed two publicly available, de-identified datasets: the Alzheimer's Disease Neuroimaging Initiative (ADNI) dataset, accessible via the LONI Image and Data Archive (IDA) at: https://adni.loni.usc.edu/data-samples/adnidata/ (Accession number: sa000002 on NIAGADS) and the Australian Imaging Biomarkers and Lifestyle Study of Ageing (AIBL) dataset, also available through the LONI IDA platform (project “AIBL”) at: https://ida.loni.usc.edu/collaboration/access/appApply.jsp?project=AIBL.
